# Medical physicists should meet with patients as part of the initial consult

**DOI:** 10.1002/acm2.12305

**Published:** 2018-03-14

**Authors:** Bradley W. Schuller, Kristi R. G. Hendrickson, Yi Rong

**Affiliations:** ^1^ Department of Radiation Oncology McKee Medical Center Banner Health Loveland CO USA; ^2^ Department of Radiation Oncology University of Washington School of Medicine Seattle WA USA; ^3^ Department of Radiation Oncology University of California Davis Comprehensive Cancer Center Sacramento CA USA

## 1 INTRODUCTION

These days cancer patients who have been advised to consult a radiation oncologist are generally very Internet savvy, and they are highly likely to go online and search for the “best” doctor, the “best” cancer clinic, the “best” treatment regimen, and/or the “best” available technology for their specific disease. Despite all of this Internet access and searching strategies, it is unlikely that they will consider searching for the “best” and most qualified physicists (or physics team). Frankly, most of the public probably don't even realize the existence of medical physicists, not to mention the importance of our responsibilities in working with the radiation oncologists to provide high quality, reliable, and safe radiation therapy. As medical physicists and dosimetrists, we work with nurses, therapists, physicians, and a wide range of professionals for the care of our patients. However, since our work is largely technical and performed without patients' present (e.g., treatment planning on computers and patient‐specific quality assurance measurements on phantoms), we might be the only team members with zero direct contact with the patients. In an effort to increase the awareness of our profession and substantial role in the clinic, the AAPM Public Education Committee has been making efforts to promote public education in matters pertaining to medical physics. What more can we do? Well, would it be a good idea to increase medical physicists’ roles in patients’ consults? For this debate, we have Dr. Brad Schuller arguing for the topic that *Medical physicists should meet with patients as part of the initial consult,* and we have Dr. Kristi Hendrickson arguing against it.

Dr. Brad Schuller received his PhD in radiation biophysics from the Department of Nuclear Science and Engineering at MIT in 2007. He then completed his postdoctoral training in therapeutic medical physics at the Massachusetts General Hospital and Boston Medical Center. He currently works for Banner Health at McKee Medical Center in Loveland, CO and is board certified by the ABR. Dr. Schuller's current research focuses on prospective risk management and exploring new roles for medical physicists in clinical practice. He is a member of AAPM and ASTRO and serves on several AAPM committees.

Dr. Kristi Hendrickson is currently an Assistant Professor of Medical Physics at the University of Washington in Seattle. She is the Director of the Medical Physics Residency Program in Therapy Physics at UWMC, which includes four total residents and 17 physics faculty mentors. Her education interests focus on medical physics residency training. She is interested in curriculum development and sharing those ideas with other institutions and programs, as evidenced in her publication and sessions created for the annual AAPM meetings. Her research interests include bioinformatics, SBRT, functional imaging, and neutron therapy. Her current AAPM committee involvement includes the Women's Professional Subcommittee and the Medical Physics Residency Training and Promotion Subcommittee. She previously served as on the course director team of the 2014 AAPM Summer School on SRS/SBRT/SABR.

## 2 OPENING STATEMENTS

### 2.1 Bradley W. Schuller, PhD

The rapidly changing healthcare environment has placed pressure on the medical physics community to define new areas of professional growth and demonstrated value. The recent AAPM initiative called, “MedPhys 3.0” (https://www.aapm.org/MedPhys30/), aims to “redefine and reinvigorate the role of physics in modern medicine”, and it calls on the community to utilize our technical expertise to increase visibility and expand into new areas of practice. Some opportunities for growth and expansion may exist outside of traditional medical physics practice and could place the clinical physicist in a more direct and collaborative role in clinical care with both the patient and physician. One such opportunity is for clinical medical physicists to meet with every patient during the initial consult to serve as an information resource and a guide through the complex treatment process. With all of the remarkable advancements in modern cancer care leading to better outcomes and increased survival with fewer side effects, the patient care pathway can be fragmented, confusing and difficult to navigate. The clinical medical physicist is ideally placed to help mitigate some of these difficulties, and along the way, help to establish trust, provide information, and reduce anxiety for the patient.

Let us first take a closer look at the current environment patients face today. Patients are asked to navigate a complicated, multidisciplinary landscape where they are expected to comprehend the information they need to make appropriate decisions about their care. They may encounter a myriad of imaging tests, surgical consults, medical and radiation oncology consults, pathology reports, and in many cases, holistic and alternative care options. When encountering each of these specialties, the patient will have to manage potentially confusing medical jargon, acronyms, cancer staging, complicated treatment concepts (e.g., genetic testing), and various treatment options that might elicit fear and anxiety. Radiation therapy has the potential of being a substantial source of anxiety, which largely stems from the general public's lack of understanding of radiation's role in medicine coupled with sensationalized media reports about nuclear weapons and radiation accidents.^1^ Therefore, communicating the risks vs. benefits of medical radiation may be ineffective or complicated by patient fear, and this could negatively impact the patient's decision making process.^1^ Outside of the clinical setting, patients have increasing access to online medical information that can assist with medical education and decision making. However, recent reports have indicated that online patient education materials deviate from NIH and AMA recommendations for levels of complexity and readability. Prabhu et al. and Rosenberg et al. recently ran independent studies where online patient education materials from major professional websites and academic radiation oncology departments were evaluated for readability at the recommended reading level. They both found that online education materials are written to a collegiate reading level, which far exceeds the recommended middle‐school reading level.^2^, ^3^ All of these factors have the potential of increasing patient distress, and this has been shown to lead to decreased survival following cancer therapy.^4^

What can medical physicists do to help? We are uniquely positioned to serve as an information resource for patients during a formalized meeting at the initial consult. First, the clinical medical physicist must establish an individual relationship with each patient. This will establish trust that their radiation treatment is being managed by a physicist who is specifically trained in the medical application of science and technology and has advanced knowledge in the use of medical radiation. As a result, the patient will identify the medical physicist as the technical authority and information resource, and as the relationship develops, the medical physicist will serve as a guide to help the patient navigate the technical aspects of their care. This new role can contribute to a patient's increased understanding, reduced anxiety, and increased satisfaction with their healthcare experience.

Atwood et al. have invited clinical medical physicists to reject the notion that we should only work behind the scenes, and they urge the community to establish new roles in direct patient care.^5^ As a result, we will have increased visibility in front of the patient leading to increased overall visibility to the rest of the clinical staff, hospital administration, and the general public. These are the keys to ensuring a robust and enduring future for medical physicists in a rapidly changing healthcare environment. Clinical medical physicists make an intentional choice to not only be scientists in medicine, but to practice medicine itself, and if we take steps to emphasize the “medical” component of medical physics in our clinical practice, we will unlock new and rewarding roles in patient care.

### 2.2 Kristi R. G. Hendrickson, PhD

The practice of medical physics is changing rapidly, and radiotherapy is increasingly technological. Safety and technological details of radiotherapy treatments are often concerns for patients considering or being advised to receive radiotherapy. Media reports on radiotherapy accidents may increase those concerns for prospective patients and their advocates.

The initial patient consult is the first direct meeting space between the patient and their attending radiation oncologist, typically with patient family and supporting friends present. Radiation treatment options and recommendations, possible side effects, and other concerns related to making the decision whether to proceed with radiotherapy are discussed. This meeting is commonly an emotional one in which the attending is also trying to understand the patient's value system and how it may affect their treatment decisions. The radiation oncologist is a guide who is providing their expert medical advice to the patient in proposing their opinion on different courses of treatment. But the ultimate decision and choice among the treatment options presented rests with the patient.

It is not necessary or prudent for a medical physicist to be routinely present at the initial patient consult. Patients may frequently ask questions about the technology that will be used for treatment or about the safety of the procedures. But most patient questions will be answered in simple terms by their attending radiation oncologist adequate enough to satisfy the great majority of patients. If in relatively rare instances a patient's questions cannot be convincingly or reassuringly answered by the patient's attending, then it may be appropriate for the attending to request a member of the medical physics team join the patient consult meeting and answer the patient's questions.

The time commitment required for a medical physicist to routinely be present at every initial patient consult would be substantial. In my clinic, there are 1200–1500 initial patient consults on average per year or 24–30 per week. The meetings are typically 20–60 or more minutes long. Expecting a physicist to be present at each of these patient meetings would be an inappropriate utilization of a limited and expensive resource — physicist time — and would therefore add an inappropriate cost to the present medical care system. Physicists are not paid or trained to do this counseling work and should instead use their skilled labor to maximize the safety, effectiveness and efficiency of radiotherapy treatments in the myriad of ways currently performed; machine‐level QA, patient‐specific plan QA, commissioning activities, continuous safety improvement programs, education of students and residents, and research and clinical development. Our current practice is for the attending physician to page a medical physicist to join an initial patient consult when needed.

Medical physicists are not trained to interact with patients in the setting of an initial patient consult. Anecdotally, I recall attending an initial patient consult with a member of my family who was considering radiotherapy. I did ask to meet the physicist and asked several questions about their QA processes as it related to the proposed treatment. What I experienced was a “deer in the headlights” reaction and a fumbling response that did not instill in me a confidence in that clinic's radiation safety processes. The University of Washington medical physics residency program I lead has developed a rotation that requires a resident to shadow radiation oncologists for several weeks and attend new patient consults. Through this rotation, they begin to appreciate how these interactions take place and how to behave and respond in patient interactions. The learning objectives of that rotation are multifaceted and are not explicitly set to train physicists in the soft skills of patient interactions. I argue that this example of training is necessary but only a start. Further soft skills training for medical physicists would be appropriate before it could be a routine part of their clinical job to appear at initial patient consults.

## 3 REBUTTAL

### 3.1 Bradley W. Schuller, PhD

Every clinical medical physicist has likely had the opportunity to meet with a few patients through direct request from the attending radiation oncologist to help answer highly technical questions or assuage safety concerns. However, one of these requests was made directly by a patient early in my career, and that interaction has had profound, lasting impact on my current clinical practice. This patient was particularly observant, and she was well informed about what to expect from radiation therapy and the personnel involved in her care. One day, as I was walking through the waiting room, the patient caught my attention and proclaimed, “You must be the medical physicist!” She'd had direct contact with every other discipline in our clinic, and I was the last on her list. We had a wonderful conversation about the clinical medical physicist's role in patient care, and I explained everything that was happening behind the scenes to ensure the highest quality for her care. When we were finished, she asked me one simple question regarding our meeting, “Why don't you offer this to every patient?” Listening closely to patient cues will help inform future directions for clinical medical physicists that will have far reaching impact on patient care.

The added medical physics time commitment is a valid concern, especially for busy departments, but I would challenge the community to look beyond the initial difficulty of establishing a new area of practice to the positive downstream effects for the patients. The medical physics patient consults will reveal a diverse collection of patient questions that will aid future patient education efforts. The creation of tailored educational materials that address specific question types will help improve the quality of the information provided to patients. By augmenting the initial consult with the radiation oncologist, clinical medical physicists can help reduce patient anxiety by revealing the mystery of radiation delivery.

I agree with Dr. Hendrickson that lack of training for direct patient interaction is a substantial concern for our community if we are to advocate widespread expansion into this new area of practice. In our clinic, we recognized that it would require some practice for clinical medical physicists to feel comfortable distilling complex technical concepts into simpler language, and to do so with confidence. One way to gain experience doing this is to deliver educational talks to patient support groups. Many of our support groups are disease specific and actively engage the community by inviting past and present patients to gather together for communal support. These groups crave new information, especially pertaining to advances in cancer treatment and new technology. Our physics group routinely gives educational talks to our support groups to help explain the technical aspects of radiation therapy. By doing so, we have had the experience of presenting to hundreds of patients and family members, and this experience has translated directly to our medical physics patient consult program by giving us experience with not only explaining technical concepts to patients but also answering questions confidently without the “deer in the headlights” effect.

### 3.2 Kristi R. G. Hendrickson, PhD

I agree that medical physicists should use their technical expertise to increase visibility of our existing health care roles and to expand into new areas of expertise. But we need to do so as a wise investment with a purposeful payoff. Not visibility for its own sake only but designed with a clear goal such as to increase public awareness of our role in ensuring the safety of radiotherapy. Physicists cannot represent themselves as an expert in all areas of health choices for a cancer patient and instead must apply their strengths and skills in the most appropriate ways.

It is true that most of our patients do not know there are medical physicists working on their behalf, let alone know what medical physicists do. This lack of visibility could be addressed in simple and cost effective ways. Many of our clinics provide information to patients about their health care team, either through printed materials that introduce their physician, nursing team, social worker, and therapists. Or perhaps through flat panel monitors mounted in the waiting areas that scroll through images and brief information that introduces primarily the physician team but may also include other clinical health care team members. Rarely do I see the medical physics team included (actually, I also do not often see the dosimetry team included either), creating an out‐of‐sight, out‐of‐mind situation. Without added cost, the medical physics team could be included with a similar level of description on par with our physician colleagues (perhaps including professional face photos, as often included on panel monitors) to increase awareness by the patient and family that physicists are part of their care team.

Additionally or alternatively, a FAQ sheet could be created by the AAPM or customized by the individual clinic that includes an introduction to the medical physicist's clinical role and describes key safety procedures and responsibilities uniquely held by the medical physicist. These materials should be created by the medical physicist and with their expert input and written at the appropriate level of the general healthcare audience. (A recommended middle‐school level, as pointed out by Dr. Schuller.)

Without added cost to the health care system, these actions would increase visibility of medical physicists to the patient, family, and public. With this introduction, a patient or their family may be more likely to ask to meet with a physicist and have specific questions for them. That would be great! And we had better be prepared to answer those questions to avoid presenting a “deer in the headlights” look. Therefore we need to create training curricula within our residency programs and for our existing medical physics teams that will teach the necessary soft skills and explicitly train us to answer questions at the appropriate patient level.

Furthermore, we need to educate physicians, nurses, and therapists to page us when patients have questions. Anecdotally, I know that patients have more technical questions after starting their treatment or when seeing the treatment machines. At this point, they may be primarily interacting with technical staff, who might brush off the questions or not take the time to call a physicist. After we have become trained and skilled in this level of patient interaction, our colleagues need to know that we are willing and happy to answer that page and to meet with the patient.

Finally, the “Opening” argument does not address questions of the best use of time, resources, and health care dollars spent. Suggestions for changes to the clinical role of a medical physicist to include meeting with the patient at the initial patient consult must be made with a view to cost–benefit analysis impact on medical physics time and how that will translate into increased cost borne by the health care system. What tasks will the physicist no longer be doing in order to devote time to initial patient consults? How many additional physicists will need to be hired in order for them to continue fulltime clinical tasks in addition to taking on this new consulting role?

Increased public and patient awareness of medical physicists and our role in ensuring patient safety of radiotherapy treatments is important. I recognize that increased interactions with patients can be a positive, if done judiciously and properly with training.

## ACKNOWLEDGMENTS

Authors thank Dr. George A. Sandison and Dr. Stanley H. Benedict for their valuable suggestions and edits.
